# Influence of Musical Enculturation on Brain Responses to Metric Deviants

**DOI:** 10.3389/fnins.2018.00218

**Published:** 2018-04-18

**Authors:** Niels T. Haumann, Peter Vuust, Freja Bertelsen, Eduardo A. Garza-Villarreal

**Affiliations:** ^1^Department of Aesthetics and Communication (Musicology), Faculty of Arts, Aarhus University, Aarhus, Denmark; ^2^Department of Clinical Medicine, Center for Music in the Brain, Royal Academy of Music, Aarhus University, Aarhus, Denmark; ^3^Center of Functionally Integrative Neuroscience, Aarhus University, Aarhus, Denmark; ^4^Department of Nuclear Medicine and PET Centre, Aarhus University Hospital, Aarhus, Denmark; ^5^Clinical Research Division, Instituto Nacional de Psiquiatría Ramón de la Fuente Muñiz (INPRFM), Mexico City, Mexico; ^6^Department of Neurology, Faculty of Medicine and University Hospital, Universidad Autonoma de Nuevo Leon, Monterrey, Mexico

**Keywords:** mismatch negativity (MMN), meter, rhythm, music, culture, MEG

## Abstract

The ability to recognize metric accents is fundamental in both music and language perception. It has been suggested that music listeners prefer rhythms that follow simple binary meters, which are common in Western music. This means that listeners expect odd-numbered beats to be strong and even-numbered beats to be weak. In support of this, studies have shown that listeners exposed to Western music show stronger novelty and incongruity related P3 and irregularity detection related mismatch negativity (MMN) brain responses to attenuated odd- than attenuated even-numbered metric positions. Furthermore, behavioral evidence suggests that music listeners' preferences can be changed by long-term exposure to non-Western rhythms and meters, e.g., by listening to African or Balkan music. In our study, we investigated whether it might be possible to measure effects of music enculturation on neural responses to attenuated tones on specific metric positions. We compared the magnetic mismatch negativity (MMNm) to attenuated beats in a “Western group” of listeners (*n* = 12) mainly exposed to Western music and a “Bicultural group” of listeners (*n* = 13) exposed for at least 1 year to both Sub-Saharan African music in addition to Western music. We found that in the “Western group” the MMNm was higher in amplitude to deviant tones on odd compared to even metric positions, but not in the “Bicultural group.” In support of this finding, there was also a trend of the “Western group” to rate omitted beats as more surprising on odd than even metric positions, whereas the “Bicultural group” seemed to discriminate less between metric positions in terms of surprise ratings. Also, we observed that the overall latency of the MMNm was significantly shorter in the Bicultural group compared to the Western group. These effects were not biased by possible differences in rhythm perception ability or music training, measured with the Musical Ear Test (MET). Furthermore, source localization analyses suggest that auditory, inferior temporal, sensory-motor, superior frontal, and parahippocampal regions might be involved in eliciting the MMNm to the metric deviants. These findings suggest that effects of music enculturation can be measured on MMNm responses to attenuated tones on specific metric positions.

## Introduction

Music is ubiquitous in human cultures (Molnar-Szakacs and Overy, 2006) and is partly defined by the rhythms, the structured patterns of sound inter-onset-intervals (between 50 and 2,000 ms), and the meter, comprising regular sequences of beats (London, 2001). The meter consists of patterns of strongly and weakly accented beats, which are organized in hierarchical levels, with slower levels at the top of the hierarchy and faster levels at the bottom (Large and Jones, 1999; Temperley, 2000). The beat can be accented by different means, for example by an increase in intensity, duration or by a change in pitch or timbre. Perceiving a musical meter does not exclusively require the presence of regularly accented beats, as the perceived meter is simultaneously based on an internal model of expectations in the listener (Palmer and Krumhansl, 1990; Temperley, 2000). This means that in cases where it is not possible to hear regular metric accents from the objective sound sequence, certain metric accents can still be expected by the listener as a consequence of lifetime exposure to metric structure (Palmer and Krumhansl, 1990; Temperley, 2000). This has been supported by behavioral and EEG experiments showing that attenuated sounds are perceived more unexpected at certain metric positions (Bolton, 1894; Temperley, 1963; Povel and Okkerman, 1981; Parncutt, 1994; Brochard et al., 2003; Potter et al., 2009).

The ability to perceive rhythm and meter in music is affected by musical training (Wallentin et al., 2010), and this could also be true for passive lifetime listening in a certain musical culture. The term “musical enculturation,” based on the recent “cultural distance hypothesis” (see Demorest and Morrison, 2016), refers to the lifetime exposure to similar musical structures within the same “culture” (Demorest and Morrison, 2016). For example, although a variety of different meters are applied in so-called Western music of Europe and North America from the Vienna-Classical era in the 1700's and until today, binary meters (based on 2 or 4 beats) seem to be the most frequently applied across individual pieces of Western music, and this meter is thus prominent. In contrast to Western music, more Sub-Saharan African music is considered to be populated by relatively complex meters. Ethnomusicologists have suggested that contrametric music (accents are placed irregularly), is common in Central African music (Kauffman, 1980; Arom, 1989; Magill and Pressing, 1997; Temperley, 2000; Agawu, 2006). The popular West African rhythm called “the standard pattern” is an asymmetrically spaced pattern of 2 + 3 beats that is similar to a typical Western 12/8 time signature, but contradicts regular metric accentuation on (4, 3, or 2) evenly spaced beats (Kauffman, 1980; Arom, 1989). Furthermore, complex meters are applied in music of other cultures such as Balkan music, where meters often consists of 7, 11 or 13 beats, or additive meters with combinations of smaller metric sequences consisting of variable numbers of beats (Goldberg, 2012). North-American 6-month-old children can detect changes in Western binary meters (simple) and Middle Eastern additive meters (complex) equally well (e.g., consisting of accentual patterns on every 2+2+3 or 3+2+2 beats), whereas at an age of 1-year and in adulthood Western listeners appear to be biased toward better detecting changes in the context of Western binary meters (Hannon and Trehub, 2005a,b; Hannon et al., 2012a; Kalender et al., 2013; Roncaglia-Denissen et al., 2013). Studies have shown that personal preferences for particular meters can be modified by short-term exposure to binary or ternary meters in infants (Phillips-Silver and Trainor, 2005) and by long-term exposure to music with more complex metric structures, such as African or Balkan musical culture in adults (Hannon et al., 2012b).

Recent studies have investigated the effects of enculturation to music of several cultures on brain function in relation to melody perception, suggesting influences of musical enculturation on brain responses to melodies (Neuhaus, 2003; Nan et al., 2006, 2008; Demorest et al., 2009; Wong et al., 2011; Matsunaga et al., 2012). With fMRI it was found that listening to culturally unfamiliar melodies showed increased blood-oxygen-level-dependent (BOLD) signal in attention related mid and right frontal regions in comparison to when listening to culturally familiar melodies (Nan et al., 2008; Demorest et al., 2009). Similarly, an EEG study found that the amplitude of the novelty and incongruence related P3 component was larger in German musicians when they listened to unfamiliar tones in Thai scales when compared to familiar Western major-mode scales, whereas no difference was observed in Indian musicians, for whom the Thai scales were structurally similar to familiar Indian scales (Neuhaus, 2003). In contrast, another fMRI study found that the BOLD signal in the auditory cortex decreased in Western listeners when they listened to Indian music compared to Western melodies, whereas no comparable decrease was observed in a bicultural group exposed to both Indian and Western music (Wong et al., 2011). With respect to meter, Brochard and colleagues using EEG showed that the brains of Western music listeners responded to −4 dB attenuated deviant beats with higher amplitude P3 components when the deviant tones occurred on “odd-numbered” positions compared to “even-numbered” metric positions (Brochard et al., 2003; Potter et al., 2009), supporting the hypothesis about an internal meter related to music enculturation. This finding is consistent with the frequent use of binary meters in Western classical music (Fraisse, 1978; Huron, 2006, p. 195). However, it is uncertain whether being exposed to culture-specific rhythms and meters also modulates other event-related potentials such as the faster and preattentive Mismatch negativity (MMN) response compared to the slower and attentive P3 component. MMN responses to musical culture deviations may suggest a highly ingrained internal model of meter related to long-term exposure.

The MMN is a fast preattentive negative deflection event-related potential (ERP) and magnetic field counter part (ERF) occurring c. 100-250 ms after a stimulus that deviates from previous stimuli, measured with EEG and its magnetic equivalent MMNm measured MEG (Näätänen et al., 2001, 2007, 2010). From here on we will refer to both the electric and magnetic mismatch negativity as MMN. Several studies have shown that irregularities in music elicit MMN (Tervaniemi, 2001; Trainor et al., 2002; Pantev et al., 2003; Tervaniemi and Huotilainen, 2003; Fujioka et al., 2004, 2005; Tubau et al., 2007; Vuust et al., 2009; Garza-Villarreal et al., 2011; Lappe et al., 2013; Bouwer et al., 2014; Hove et al., 2014; Putkinen et al., 2014; Tervaniemi et al., 2014). The MMN does not require attention to be elicited (Näätänen, 1988; Sadia et al., 2013) and it reflects errors in the brain's prediction of the stimuli in the environment (Friston, 2005; Strelnikov, 2007; Garrido et al., 2009; Vuust et al., 2009; Bendixen et al., 2012; Rohrmeier and Koelsch, 2012). It initiates fast involuntary attention shifts (Näätänen, 1988, 2001; Kujala et al., 2007; Näätänen et al., 2007, 2010), and is involved in implicit learning (Friston, 2005; Strelnikov, 2007). The MMN is dependent on memory and it is usually higher in amplitude and faster in latency the more a stimulus deviates from a pattern that is learned to occur frequently in a particular environment, such as a musical pattern in a specific genre (Brattico et al., 2001, 2006; Näätänen et al., 2001, 2005, 2007, 2010; Jones, 2002; Vuust et al., 2005, 2009, 2012; Kujala et al., 2007; Kujala and Näätänen, 2010; Lappe et al., 2013; Paavilainen, 2013; Hove et al., 2014). Also, the MMN has been found in response to deviations from binary and ternary meter accents in Western music (Vuust et al., 2005; Martin et al., 2007; Honing et al., 2009; Geiser et al., 2010; Bouwer et al., 2014). However, it has not been described how the MMN response depends on familiarizing with musical structures of particular cultural environments by comparing different cultural groups (but see Putkinen et al., 2014; Tervaniemi et al., 2014). Therefore, the MMN is an ideal component to study rhythm and meter in the context of musical enculturation.

By means of MEG, we investigated whether music listeners' expectations toward binary meters could be affected by enculturation effects reflected in the amplitude of the MMNm component. We expected to replicate previous findings (Vuust et al., 2005; Martin et al., 2007; Honing and Ladinig, 2009; Geiser et al., 2010; Bouwer et al., 2014) that listeners exposed to Western music show an MMN with higher amplitude in response to −4 dB softer odd-numbered tones in comparison to −4 dB softer even-numbered tones. If binary meter expectations were subject to musical enculturation, we expected this difference in response to softer odd and even tones to be decreased in listeners with bicultural exposure to both Western and Sub-Saharan African music. Further, we wanted to investigate whether a metric derived MMN would originate in the auditory cortex, and if the inferior temporal lobe, motor cortex, and premotor cortex, found in previous MRI studies on listeners' rhythmic abilities (Chakravarty and Vuust, 2009; Bailey et al., 2014), may be involved.

## Methods

### Ethics statement

This study was carried out in accordance with the recommendations of the Ethics Committee of Region Mid-Jutland in Denmark with written informed consent from all subjects. All subjects gave written informed consent in accordance with the Declaration of Helsinki. The protocol was approved by the Ethics Committee of Region Mid-Jutland in Denmark.

### Participants

Twenty-five participants (13 females) of average age 27 years (range: 18–39 years) were recruited for the study, which was conducted at the Danish Neuroscience Center, Aarhus, Denmark. The inclusion criteria for participation were: (a) to have an interest in music, (b) to have normal hearing and be healthy, (c) to be of an age between 18 and 39 years, and (d) to be right-handed. The exclusion criteria were: (a) use of strong medicine or psychoactive drugs, (b) to have un-removable metal parts in the body, (c) to suffer from claustrophobia, and (d) have a head circumference of larger than 60 cm (due to the size of the MEG helmet and MRI head coil). Since our purpose with the study was to find two groups with distinct listening habits (a Western and a bicultural music listening group), the participants filled out an online questionnaire to enable us to analyze their amount of exposure to particular music genres of different cultures.

We organized our sample into two musical culture groups: a Bicultural group with 13 (6 females) participants who reported that during their life they had listened to music from both African (for 1–31 years, average 12.3 years) and Western cultures, and a Western group consisting of 12 (7 females) participants who reported that they during their life had listened mainly to music of Western cultures (for 18–36 years, average 26.5 years) and no African cultures. The two groups were also exposed to music of other non-Western cultures during their life span, and there was a trend in the Bicultural group to be exposed to more music of other non-Western cultures (than African music) compared to the Western group, which was in particular Balkan music (Table 1). Both groups had on average lived about 2/3 of their lives in Denmark, the most frequent native language was Danish, and the groups did not differ significantly on these two factors (Table 1). In the Bicultural group the average age was 27.3 years (range: 19–39 years) and in the Western group 26.5 years (range: 18–36), and the two groups were balanced with regard to age (Table 1). To ensure that the groups were equally exposed to music, they were also balanced with regard to the Goldsmith's Musical Sophistication Index factor 1 (G-MSI 1), “personal importance of music” (i.e., how many hours per day the participant listens attentively to music; Müllensiefen et al., 2011). For the present study we did not distinguish between music exposure acquired through passive listening or actively playing an instrument. However, since rhythm perception ability could influence the MMNm, both groups were matched on the Musical Ear Test (MET) for rhythm perception scores (Wallentin et al., 2010), a discrimination task involving judgments about whether pairs of rhythmic patterns are same or different, and which is able to distinguish between the differences in rhythm perception ability of non-musicians, amateur musicians, and professional musicians. The MET might be biased toward testing rhythm perception ability in Western music listeners and not necessarily non-Western music listeners; though, we assumed the test would still be valid, since all participants were exposed to Western music. Furthermore, 14 participants (seven from each group) volunteered having T-1 MRI scans acquired, which were applied subsequently for source localization analysis.

**Table 1:** Match of variables between the Bicultural group and Western group.

	**Bicultural group**		**Western group**		**Comparison of groups**		
**Variable**	***n* (%)**	**mean (range)**	***n* (%)**	**mean (range)**	***χ^2^***	***t***	***p***
**MEG STUDY**							
Years exposure to Sub-Saharan African music	–	9.9 (1–31)	–	0 (0)	–	4.40	<0.01
Years exposure to other non-Western music	–	11.3 (0–30)	–	5.2 (0–20)	–	1.69	0.10
Years exposure to Western music	–	25.6 (17–39)	–	27.1 (18–36)	–	0.66	0.51
Native language is Danish	7 (54%)	–	9 (75%)	–	1.21	–	0.27
Years living in Denmark	–	17.3 (1–33)	–	18.9 (1–30)	–	0.42	0.68
Years living in other countries	–	9.0 (0–30)	–	7.3 (0–32)	–	0.38	0.71
Age	–	26.5 (19–39)	–	27.3 (18–36)	–	0.67	0.67
Sex (females)	6 (46%)	–	7 (58%)	–	0.37	–	0.54
Goldsmith's Musical Sophistication Index factor 1	–	76.6 (58–96)	–	76.7 (43–99)	–	0.02	0.99
Musical Ear Test for rhythm	–	42.4 (31–48)	–	39.9 (33–48)	–	1.26	0.22
**WEB-BASED SURVEY**							
Years exposure to Sub-Saharan African music	–	15.0 (1–39)	–	0 (0)	–	8.35	<0.01
Years exposure to other non-Western music	–	13.6 (0–39)	–	5.4 (0–31)	–	3.30	<0.01
Years exposure to Western music	–	28.2 (17–59)	–	28.0 (18–65)	–	0.09	0.93
Age	–	28.6 (18–59)	–	28.0 (18–65)	–	0.30	0.76
Sex (females)	19 (54%)	–	26 (63%)	–	0.42	–	0.42
Goldsmith's Musical Sophistication Index factor 1	–	77.8 (58–101)	–	74.8 (39–102)	–	1.00	0.32

*n shows number of participants with percentage of participants in the group in parenthesis. Continuous variables are tested statistically with independent samples t-tests and categorical variables with the Pearson chi-square χ^2^*.

Furthermore, 76 healthy participants with an average age of 28.3 (range 18–65) were recruited to rate the surprise of omitted beats in drum sequences in a web-based survey, including the participants in the MEG study. The criteria for participation were to have an interest in music and to be able to hear the stimuli clearly on the computer. By following the same procedure as for the MEG study, the 76 participants were split into a Bicultural group with 35 participants [average age 28.6 years (range: 18–59 years)] and Western group with 41 participants [average age 28.0 years (range: 18–65 years)]. The MET test for rhythm perception is restricted for research and student assessment at music schools, and distribution of the test was prohibited for the web-based survey. However, also the Bicultural and Western groups in the web-based survey were balanced with regards to age, sex, and the G-MSI 1 (Table 1). The Bicultural group in the web-based survey tended to be exposed more to other non-Western music, which was most frequently Latin American music, compared to the Western group (Table 1).

### Data acquisition

The MEG data was recorded in a magnetically shielded room with an Elekta Neuromag™ TRIUX 306 channel MEG system (Elekta Oy, Helsinki, Finland) with 102 sensor elements comprised of 102 orthogonal pairs of two planar gradiometer sensors and 102 axial magnetometer sensors. The data were recorded at a sample rate of 1 kHz with 32 bits. Immediately before each recording was started, trapped flux in any of the sensors were released by heating and re-cooling the sensor until all sensor channels had a noise value of maximum 5 fT/$\sqrt{Hz}$ for magnetometers and 5 fT/(cm$\sqrt{Hz}$) for gradiometers.

Sound stimuli and trigger signals were provided with E-Prime 2.0 stimulation software (Psychology Software Tools, Sharpsburg, Pennsylvania, U.S.A.). After MEG data acquisition, the participants were tested with the MET for rhythm perception in a nearby silent room. Finally, anatomical MRI data was acquired using a 3T Trio system (Siemens, Erlangen, Germany) with a 3D T1-weighted (T1w) MPRAGE sequence (sagittal plane orientation, voxel size = 1 × 1 × 1 mm, number of slices = 176, ascending slice order, ascending view order, TR = 2,420 ms, TE = 3.7 ms, flip angle = 9 degrees, TI = 960 ms, FOV = 256 mm, matrix = 256 × 256, slice thickness = 1 mm, 32 channel head coil).

### Stimuli and paradigm

**Figure 1:**
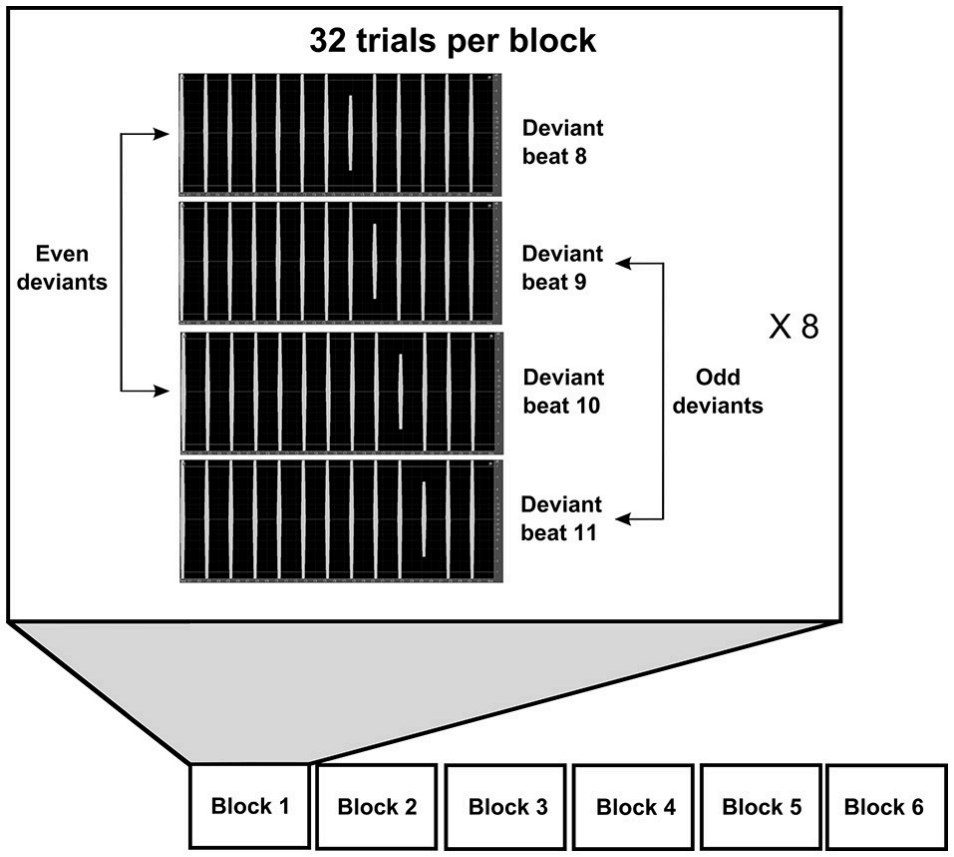
Illustration of the stimuli and paradigm. The stimuli consist of patterns of 13–16 consecutive sinus tones with even-numbered beats 8 or 10 or odd-numbered beats 9 or 11 attenuated by −4 dB. The participants listened to six blocks of stimuli, each with 32 trials.

We applied an adaptation of an ambiguous metric oddball paradigm with 3,250–4,000 ms tone sequences (Brochard et al., 2003; Supplemental Materials: Audio examples). 50% of the stimuli consisted of sequences with 13 tones, 25% of long sequences with 15 tones and 25% of long sequences with 16 tones. All sequences contained sinusoidal tones of duration 50 ms, with 10 ms fade-in and fade-out, and with an interonset interval of 250 ms (Figure 1). We applied this interonset interval both to optimize the paradigm for measuring the MMNm and to make sure the stimuli did not indicate any particular music genre, since this interonset interval is frequently applied in both Western and Sub-Saharan African music (Temperley, 2000). Each tone delineates a metric position number, and the tone sequences were played with equal sound amplitude and equal interonset interval to avoid that any metric accent could be perceived in the stimuli (i.e., the stimuli would not motivate the listeners to interpret the sequences in accordance with any particular musical meter; Brochard et al., 2003). Deviant tones were reduced in sound intensity by −4 dB, and they occurred in 96 trials on even metric positions 8 or 10 (48 trials on each even position) and in 96 trials on odd metric positions 9 or 11 (48 trials on each odd position; cf. Brochard et al., 2003). From here on we will refer to these deviant conditions as the even deviants and odd deviants, respectively. To isolate the elicited MMNm response from the brain responses to the standard tones we defined beat positions 4, 5, 6, and 7 as the standards. We wanted to make participants focus their attention on the sound stimuli as if they were in a context of listening to music and to reduce sleepiness in the participants to limit alpha waves. Therefore, we instructed the participants to detect and respond to an additional late pitch deviant inserted randomly on metric position number 13–16 (with equal probability for each of these metric positions). The pitch change appeared in 1/24 trials and consisted of a 200 cents change (i.e., by a factor of 2^(200/1,200)^ frequency in Hz) up in 1/48 trials and down in 1/48 trials. Also, to limit phase synchrony between the event-related fields and possible alpha waves, we added a break with a random jittered duration between each sequence, with 32 different random but evenly distributed break durations between 690 and 1,310 ms (average duration 1,000 ms). The stimuli were presented in 6 blocks with 32 trials in random order (cf. Brochard et al., 2003). The total duration of the auditory stimuli was 13 min and 48 s. The stimuli were recorded and edited with Adobe Audition 3.0 (Adobe Systems Incorporated, San Jose, CA, U.S.A.) in 44.1 kHz mono wave file format.

In the web-based surprise rating task the same experimental paradigm was applied, except that the tone sequences consisted of just 12 tones played with drum sounds. Also, as an additional control, a condition with the 12 tones presented without any omitted beats was applied. All tone sequences were first played with a Ghanaian West-African drum sound and secondly with a conga sound. The African drum and conga sounds were adjusted in timbre, amplitude, and duration to have matching peak frequency of 440 Hz, peak frequency amplitude level of −24 dB, and duration of 250 ms. Participants were asked to rate the level of surprise (Koelsch et al., 2008) in response to the last part of the rhythm on a 7 point Likert scale (0–6 points), on which the number 0 indicated that the last part of the rhythm was predictable, 3 that it was somewhat surprising, and 6 that it was surprising. Surprise ratings have been found to reflect the degree of unexpectedness (Koelsch et al., 2008). In this study, if a beat was omitted on a metric position, this would be perceived as more surprising for a metric position expected to be accented compared to a metric position that was not expected to be accented.

### Participant preparation

Before the MEG recording, all participants were informed about the procedure of the experiment. First, they were explained that they will be hearing some rhythms, and they were asked to sit as still as possible while listening to the rhythms. For the attention task the participants were instructed to push a button when a higher or lower tone occurred. Also, they were instructed to have a ~1 min break (occurring between the 6 stimuli blocks, each lasting 2 min and 18 s), until they by pressing a button decided to continue listening to the stimuli. The participants were explained that they were always able to communicate with the experimenters through a monitor camera and auditory communication system. These instructions were provided both before and repeated after the participants were positioned inside the MEG system.

For continuous tracking of head position in relation to the MEG sensor array the participants had 5 head position indicator (HPI) coils attached, one on the left, mid and right side of the upper forehead, and one behind the left and right. The left preauricular, nasion and right preauricular fiducial landmarks and ca. 50–70 equally distributed points on the scalp were measured using a (3D) digitizer to allow alignment of MEG and MRI data. 2 vertical EOG electrodes, 2 horizontal EOG electrodes, 1 reference electrode above the nose and 1 ground electrode attached on the arm were applied to measure eye blinks. The MEG system gantry was placed in the upright position, and participants were seated maximum 60 mm below the MEG sensors. The sound was delivered through non-magnetic plastic tubes with attached earpieces, and the sound volume was adjusted to 60 dB above the just audible sound level for each participant.

### Data pre-processing

To reduce electromagnetic noise originating from outside and near the participant's head and compensate for head movements we applied Elekta Neuromag™ MaxFilter 2.2 Temporal Signal Space Separation (tSSS) and movement compensation (Taulu and Simola, 2006; Taulu and Hari, 2009) on all data. We used the default inside expansion order of 8, outside expansion order of 3, optimization of both inside and outside bases, default subspace correlation limit of 0.980, default raw data buffer length of 10 s, and the data were down sampled from 1 kHz to 250 Hz.

The data were further processed with FieldTrip version r8165, open source toolbox for Matlab (Donders Institute for Brain, Cognition and Behaviour/Max Planck Institute, Nijmegen, the Netherlands; Oostenveld et al., 2011) in Matlab R2013b (MathWorks, Natick, Massachusetts). To reduce muscular artifacts a low pass filter at 25 Hz was applied, and to reduce slower oscillations caused by movements and baseline drifts a high pass filter at 1 Hz was applied. Next, the data from the magnetometer and gradiometer channels were visually inspected and corrected for eye movement artifacts with Independent Component Analysis (ICA). The eye movement artifacts were reduced by subtracting the back-projected channel data for the strongest vertical eye blink component and the horizontal eye saccade component. Also, any influence of heart beat artifacts was reduced by removing the back-projection of the strongest heart beat component. On average 2.7 components (range: 1–3) per participant from the magnetometer channels and 2.5 components (range: 1–3) from the gradiometer channels were applied for artifact correction. Subsequently, single trial epochs were extracted using a time window of −100 to 500 ms in relation to the standard or deviant tone onset. Baselines were adjusted according to the mean waveform in the time window from −100 to 0 ms. Some trials were contaminated by alpha or mu waves, which were relatively strong in amplitude in comparison to the MMNm responses. To reduce the interference from these alpha or mu waves, trials with amplitudes exceeding a threshold of 1,500 fT for the magnetometers and 300 fT/cm for the gradiometers were rejected. After artifact rejection the average number of trials was 86 (range: 39–96) for even deviants, 86 (range: 39–96) for odd deviants, and 729 (range: 330–768) for standards.

### Analysis of event-related fields

Difference waves showing the MMNm were created by subtracting the average responses to the standards from the average responses to the deviants. Since the gradiometer sensors measure the difference in the magnetic field across two orthogonal directions, the measures from each couple of longitudinal and latitudinal gradiometer sensor were combined by applying the Pythagorean distance formula, as implemented in FieldTrip, $d=\sqrt{{\text{longitudinal}}^{2}+{\text{latitudinal}}^{2}}$.

For participant-level analysis, mean amplitudes and peak latencies of the MMNm were calculated with application of Matlab R2013b (MathWorks, Natick, Massachusetts). The mean amplitude was determined using a 30 ms time window around the peak MMNm amplitude in the channel with the strongest amplitude in the grand average across all participants and deviant conditions, which occurred at latency 168 ms in both the gradiometer channel (0242+0243) and magnetometer channel (2411). Peak latencies for each participant and deviant condition were estimated by finding the time points of the peak amplitudes within a latency range of 100–200 ms after the stimulus onset.

SPSS version 20 (IBM, Armonk, New York, USA) was applied for group-level statistics on the amplitude and latency of the MMNm. Statistical comparisons were conducted on the gradiometer sensors, since they display higher signal-to-noise ratios compared to the magnetometers (Hämäläinen et al., 1993). To further optimize the signal-to-noise ratio we obtained the average response across four combined gradiometer channels above the right hemisphere showing the strongest MMNm amplitude in the grand average (1,332+1,333, 1,132+1,133, 1,312+1,313, 1,412+1,413) and four combined gradiometer channels above the left hemisphere (0242+0243, 0442+0443, 0222+0223, 0122+0123) mirroring the positions of those in the right hemisphere. A mixed ANOVA model was applied for testing effects of *Enculturation* (Western, Bicultural), deviant *Pattern* (even, odd), a *Control* (early, late) factor for testing whether MMNm amplitude or latency differed between early deviants on metric positions 8 or 9 and late deviants on metric positions 10 or 11, and *Hemisphere* (left, right). In addition, we tested for possibly confounding variables by inserting them as covariates in the ANOVA models.

### Source localization analysis

Source localization analysis was performed with SPM8 version r6313 (Wellcome Trust Centre for Neuroimaging, London, United Kingdom) (Friston et al., 2007, 2008). First, individual head meshes consisting of 8196 vertices were constructed from the individual T1w MRI image, and the MEG and T1w MRI coordinate systems were co-registered for each participant by manually locating the left preauricular, nasion, and right preauricular fiducial landmarks. Forward lead fields were computed by applying the single-shell model for MEG (Nolte, 2003) implemented in FieldTrip. Source locations from the magnetometer data were estimated with the standard GS algorithm in SPM8 (Friston et al., 2008) for each participant with T1w MRIs (*n* = 14; 7 in the *Bicultural group*; 7 in the *Western group*). The applied source inversion algorithm is based on multiple sparse priors (MSP) consisting of patches in the cortex representing likely source locations (Friston et al., 2008; López et al., 2014). The Greedy Search (GS) inversion is applied to find a combination of currents in the cortical patches explaining most variance in the sensor array (Friston et al., 2008; López et al., 2014). Previous findings suggest that GS source inversion provide higher spatial accuracy compared to more traditional minimum-norm estimates (MNE) or low-resolution electromagnetic tomographies (LORETA) (Friston et al., 2008; López et al., 2014). Though, the GS algorithm does not provide information about source orientations, and it estimates amplitudes in a normalized arbitrary unit, however, these parameters were not of primary interest in the present study. Since the GS inversion algorithm in addition favors source location estimates that are consistent across time, we attempted to further reduce the influence of alpha or mu waves in the source estimates by constraining the solutions to a time window of interest defined as the 30 ms time window around the MMNm peak amplitude at 168 ms after the stimulus onset. The resulting individual source images show relative source power in an arbitrary unit normalized across conditions and participants. These normalized images were warped into the MNI coordinate system. Group level source statistics was performed with one-sample *t*-tests with family-wise error (FWE) correction at alpha = 0.05 to infer which voxels showed signal power differing significantly from the baseline (i.e., signal power = 0 a.u.) across participants. Additionally, Brodmann areas were estimated from the statistically significant MNI coordinates by using the MNI to Talairach conversion tool with Brodmann areas from the Yale BioImage Suite Package (Lacadie et al., 2008). 3D rendering of the source estimates was performed with MRIcroGL (Rorden et al., 2007).

### Analysis of surprise ratings

A mixed ANOVA model was applied to test the surprise ratings in the web-based survey. The model tested for effects of *Enculturation* (Western, Bicultural), deviant *Pattern* (even, odd), and *Control* (early, late) factors on the level of surprise.

## Results

The Music Ear Test (MET) for rhythm suggested a relatively high performance in rhythmic discrimination ability in all participants in the MEG study with an average score of 41.1 (range: 31–48), which corresponds to 79% (range: 60–92%) of the maximum test score of 52. There were no significant differences in rhythm perception ability between the Bicultural and Western group as measured with the MET for rhythm (Table 1).

### Effects of metric position on surprise ratings

**Figure 2:**
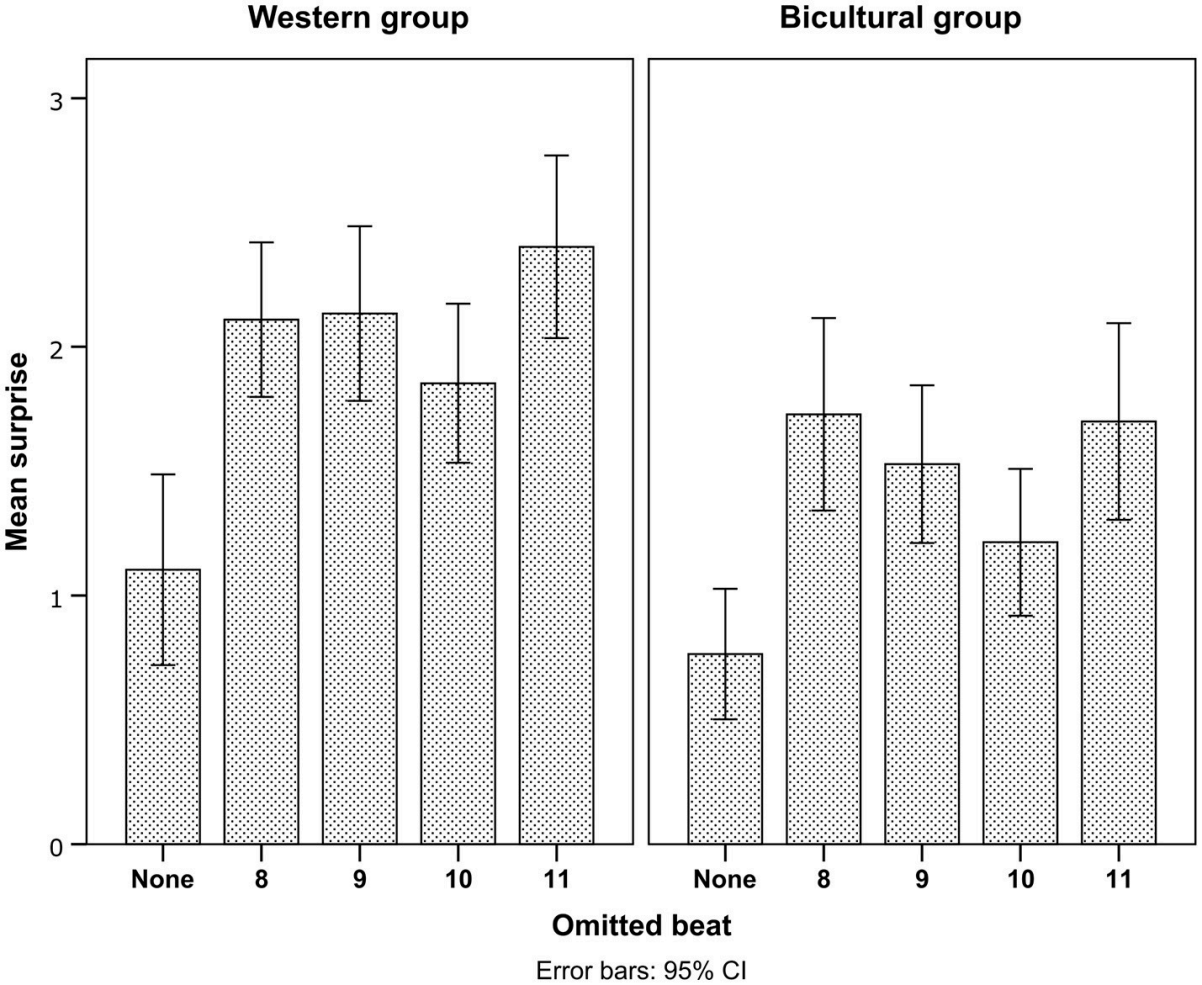
Surprise ratings for all conditions and groups. Error bars indicate 95% confidence intervals.

A main effect of *Pattern* on surprise showed that deviants on odd metric positions (9 and 11) were rated as more surprising than deviants on even metric positions (Table 2 and Figure 2). Also, the average surprise responses suggested that the Bicultural group discriminated less between deviants on odd and even metric positions (Figure 2), though in the surprise ratings we did not find statistical support for this trend in an interaction between *Enculturation* and *Pattern* (Table 2).

**Table 2:** Effects of omitted beats on surprise responses in the Bicultural and Western group.

	***df***	***df* error**	***F***	***p***	***η_*p*_*^2^**
**EFFECT ON SURPRISE**					
Enculturation	1	74	9.15	0.007^*^	0.11
Pattern (odd, even)	1	74	5.51	0.034^*^	0.07
Enculturation × Pattern	1	74	0.62	0.496	0.01
Control (early, late)	1	74	1.16	0.381	0.02
Pattern × Control	1	74	13.07	0.002^*^	0.15

df designates the degrees of freedom. Significant effects are marked by *. Estimated effect sizes are provided in partial eta squared values, $\eta_{\text{p}}^{2}$.

**Figure 3:**
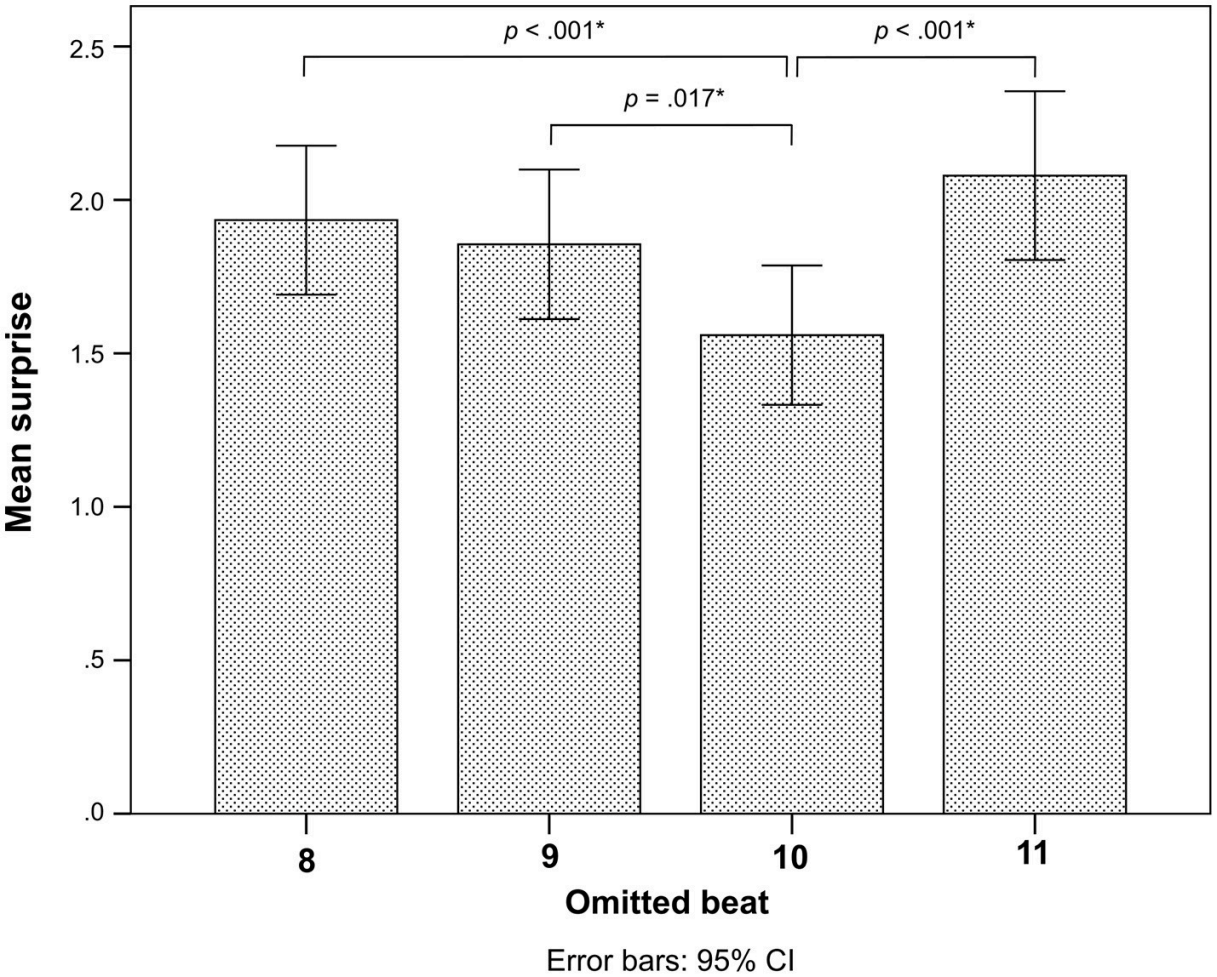
*Post-hoc* comparisons for surprise of each omitted beat position as rated by all participants. Error bars indicate 95% confidence intervals. The *p*-values are corrected for multiple comparisons with the FDR method, and the significant *p*-values are shown and marked by asterisks ^*^.

There was no difference in surprise whether the deviant occurred early or late (Table 2). However, there was a significant interaction between *Pattern* and *Control* (Table 2). *Post-hoc* comparisons indicated that this interaction was related to higher surprise responses for the even deviant on metric position 8 compared to metric position 10 (Figure 3).

### MMNm waveforms

**Figure 4:**
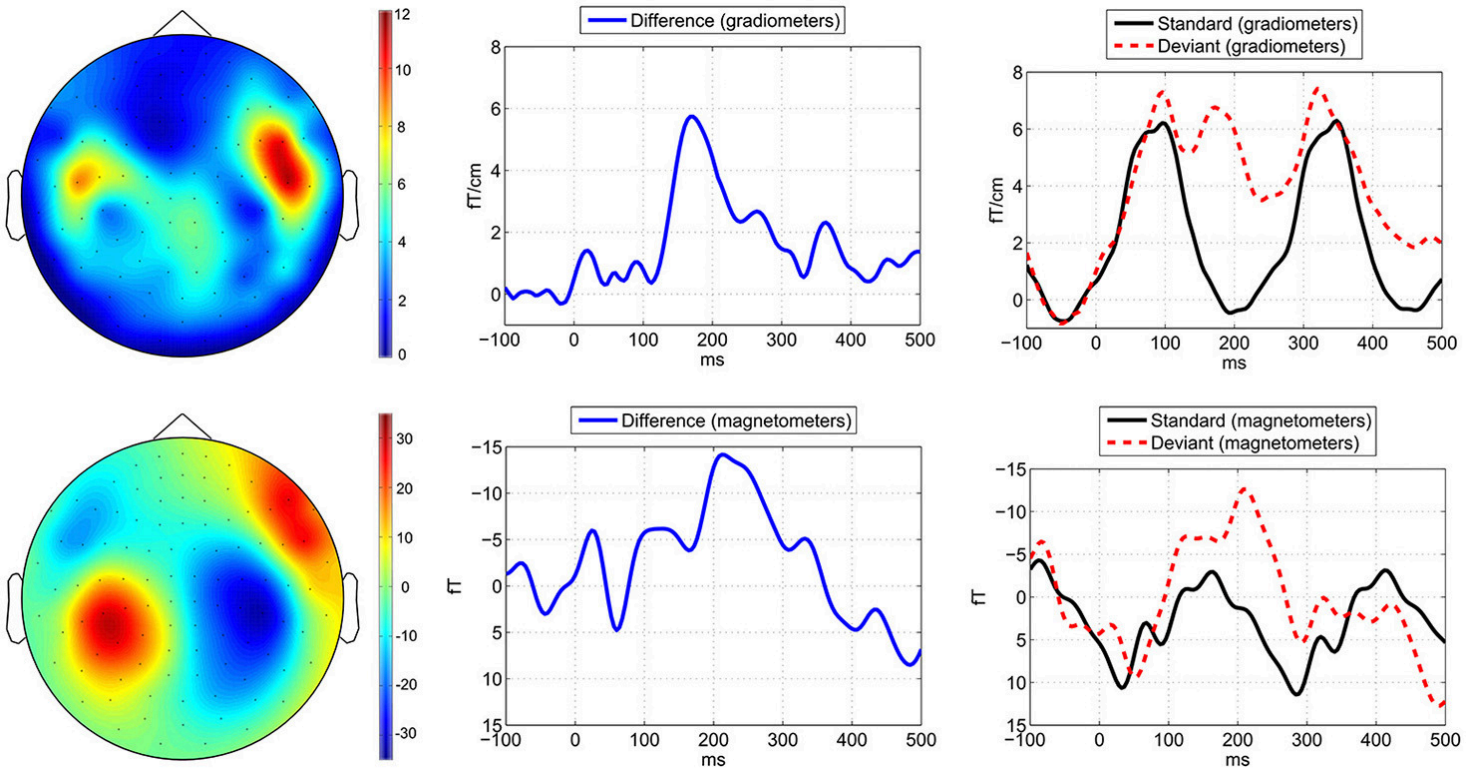
Metric deviant MMNm shown in grand averages across all participants. Average brain responses to the −4 dB deviant tones compared to the standard tones measured across the applied eight combined gradiometer channels **(top)** and eight magnetometer channels **(bottom)**. In the left side the topographies of the MMNm are visualized based on the difference waveforms by applying a time window of 30 ms around the peak MMNm latency at 168 ms. In the middle is shown the MMNm in the difference waveforms and in the right side the waveforms for the deviants (red dashed line) and standards (black solid line). The horizontal axes depict the latency in milliseconds (ms) and the vertical axes the amplitude of the magnetic field.

The recorded head positions showed that no head movements were larger than 25 mm, and therefore no additional noise should have been introduced due to the distances between heads and MEG sensors. In response to the attenuated deviant beats compared to the standard beats we observed an MMNm in the latency range between c. 100 and 200 ms in the gradiometers and magnetometers (Figure 4) in all participants. The MMNm can be seen clearly in the gradiometer channels; in the magnetometer channels it is partially distorted by alpha or mu waves.

**Figure 5:**
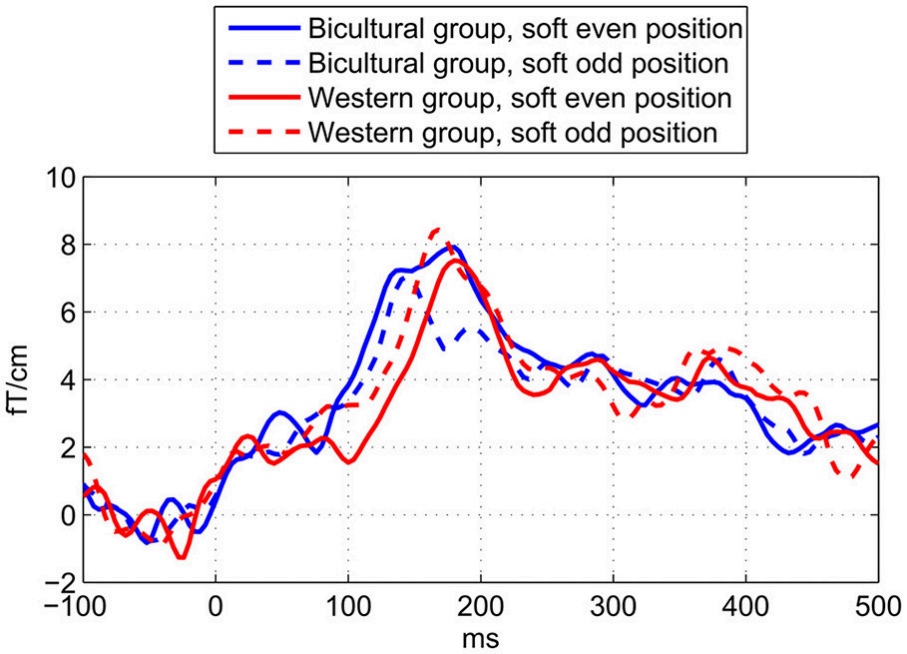
Waveforms showing enculturation effects on metric deviant MMNm. Showing the MMNm difference waveforms for the cultural groups and metric deviant positions. The MMNm amplitude in the Western group (red) is significantly stronger to the odd deviants (dashed line) than even deviants (solid line), but not in the Bicultural group (blue). Also, the latency of the MMNm is significantly shorter in the Bicultural group (blue) compared to the Western group (red). The horizontal axis shows the latency in ms. The vertical axis shows the average amplitude of the evoked magnetic field in femto-Tesla per centimeter (fT/cm) measured across the applied eight sensors above the left and right hemisphere.

As expected we found a statistically significant interaction between *Enculturation* and *Pattern* on the MMNm amplitude suggesting higher amplitude to odd deviants (mean = 13.3 fT/cm) than even deviants (mean = 12.0 fT/cm) in the Western group but not in the Bicultural group (odd deviant mean = 10.6 fT/cm; even deviant mean = 12.3 fT/cm; Figure 5 and Table 3). We did not observe any effect of the *Control* factor, which suggests that the MMNm amplitude did not differ between deviant beats on early metric positions 8 or 9 (mean = 12.5 fT/cm) compared to late metric positions 10 or 11 (mean = 12.1 fT/cm; Table 3). This supports that the Western group showed larger MMNm amplitude to odd deviants, and it shows that this effect was not caused by the fact that the odd deviants occurred later than the even deviants. We did not observe any confounding effects of MET for rhythm score [*F*_(13, 10)_ = 2.13, *p* = 0.118], G-MSI 1 score [*F*_(20, 3)_ = 2.33, *p* = 0.266], age [*F*_(13, 10)_ = 0.48, *p* = 0.894], sex [*F*_(1, 22)_ = 0.75, *p* = 0.396], or interaction between exposure to music of other non-Western cultures and *Pattern* [*F*_(1, 23)_ = 0.64, *p* = 0.43] on MMNm amplitude.

**Table 3:** Statistical results for enculturation effects on metric deviant MMNm.

	***df***	***df* error**	***F***	***p***	***$\eta_{p}^{2}$***
**EFFECT ON MMNm AMPLITUDE**					
Enculturation	1	23	0.34	0.568	0.01
Pattern (odd, even)	1	23	0.08	0.774	0.00
Enculturation × Pattern	1	23	4.70	0.041^*^	0.17
Hemisphere	1	23	0.36	0.967	0.00
Control (early, late)	1	23	0.26	0.615	0.01
Enculturation × Control	1	23	0.04	0.842	0.00
**EFFECT ON MMNm LATENCY**					
Enculturation	1	23	8.07	0.009^*^	0.26
Pattern (odd, even)	1	23	3.89	0.061^†^	0.15
Enculturation × Pattern	1	23	0.18	0.676	0.01
Hemisphere	1	23	0.20	0.656	0.01
Control (early, late)	1	23	0.95	0.340	0.04
Enculturation × Control	1	23	0.65	0.429	0.03

Effects of enculturation on MMNm amplitude and latency measured in the combined gradiometer sensors and estimated with two mixed-effect ANOVA models. df designates the degrees of freedom. Significant effects are marked by *. Trends approaching significance are marked by †. Estimated effect sizes are provided in partial eta squared values, $\eta_{\text{p}}^{2}$.

A main effect of *Enculturation* on MMNm latency showed that the MMNm to the metric deviants occurred slightly earlier in the Bicultural group (mean = 151 ms) in comparison to the Western group (mean = 163 ms; Figure 5 and Table 3). No significant interaction between *Enculturation* and *Pattern* was found for MMNm latency. Irrespective of *Enculturation*, there was a trend of the MMNm latency to be shorter for the odd (mean = 155 ms) compared to the even metric position deviants (mean = 160 ms), though the trend was not significant at the alpha threshold (Figure 5 and Table 3). Neither the MET for rhythm score [*F*_(13, 10)_ = 2.13, *p* = 0.118], G-MSI 1 score [*F*_(20, 3)_ = 2.33, *p* = 0.266], age [*F*_(13, 10)_ = 0.48, *p* = 0.894], sex [*F*_(1, 22)_ = 0.75, *p* = 0.396], or interaction between exposure to music of other non-Western cultures and *Pattern* [*F*_(1, 23)_ = 1.06, *p* = 0.314] confounded with the observed effects on MMNm latency.

### MMNm source localization

**Figure 6:**
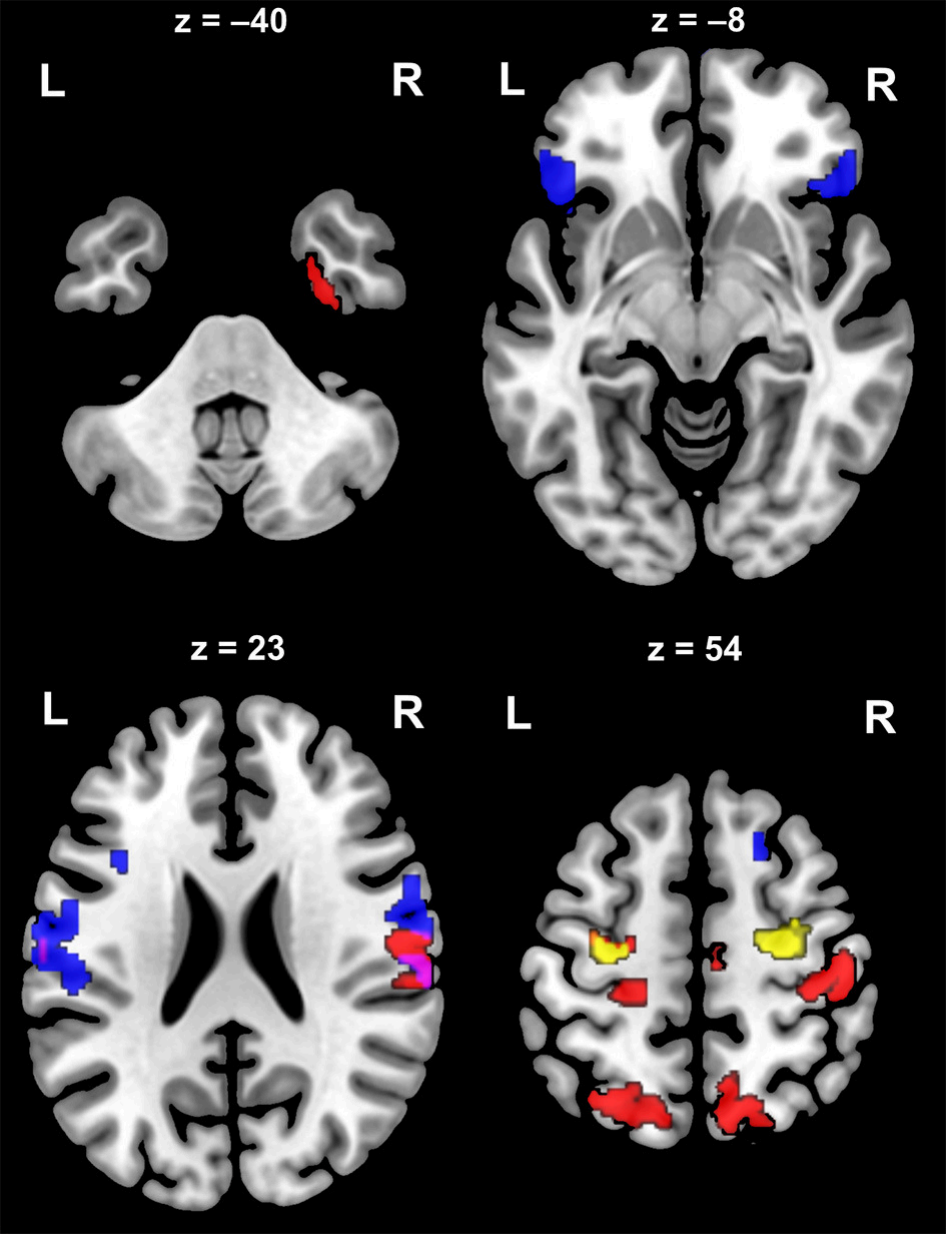
Estimated source locations of the metric deviant MMNm. Axial source images calculated created with MRIcroGL showing thresholded *t*-maps from SPM8 on MNI brain template. Source estimates of the evoked MMNm difference waveforms to the metric deviants (red), and standard tones (blue), and estimated locations where the MMNm amplitude to deviants on odd metric positions is larger in the Western group compared to the Bicultural group (yellow). The colors mark locations where comparisons of the estimates across participants satisfies the *p* < 0.05 level with Family-Wise Error correction at cluster-level, except the comparison between the groups (yellow) showing possible locations at a threshold of *p* < 0.01 without correction for multiple comparisons. Left and right hemispheres are denoted by L and R, and the numbers above the slices indicate the MNI RAS z-coordinates.

Evoked ERF (event-related field) responses to the standard tones were estimated to originate from the right auditory cortex, bilateral inferior motor cortex, frontal regions (BA40, BA44, BA45, BA47), bilateral premotor cortex/supplementary motor area (SMA), left sensory cortex, and bilateral occipital lobe (BA19) (see Table 4 and Figure 6). Estimates of the sources of the evoked MMNm responses in the difference waves suggested locations in the bilateral auditory cortex, left inferior temporal cortex (BA21), bilateral frontal cortex (BA8), bilateral premotor cortex/SMA, left motor cortex, bilateral sensory cortex, bilateral parietal cortex (BA7, BA40), right parahippocampal gyrus, and left occipital lobe (BA19) (see Table 4 and Figure 6). A *post-hoc* test suggested that the stronger MMNm amplitude to odd deviants in the Western group compared to the Bicultural group might be explained by difference in activity originating from the bilateral premotor cortex/SMA, however this was only observed without correction for multiple comparisons (see Table 4 and Figure 6).

**Table 4:** Source locations as indicated by the *t*-maps.

		**Left hemisphere**			**Right hemisphere**		
**Condition**	**Anatomical structure**	**Coordinate**	***t***	***p***	**Coordinate**	***t***	***p***
Standard	Inferior motor cortex	−60, −4, 16	11.74	<0.001	58, −4, 24	11.23	<0.001
	Inferior frontal cortex (BA47)	−46, 30, −8	7.02	<0.001	–	–	–
	Brocas area (BA45)	–	–	–	48, 28, 0	6.92	<0.001
	Parietal cortex (BA40)	–	–	–	42, −36, 48	6.82	<0.001
	Premotor cortex / SMA (BA6)	−8, −22, 74	6.42	<0.001	12, −20, 70	6.24	<0.001
	Sensory cortex	−44, −34, 48	6.39	<0.001	–	–	–
	Occipital lobe (BA19)	−44, −80, 16	6.21	0.001	50, −70, 20	5.71	0.001
	Brocas area (BA44)	−36, 14, 24	6.06	0.003	–	–	–
	Premotor cortex / SMA (BA6)	−24, −2, 64	5.51	0.006	20, 20, 52	5.88	0.001
	Auditory cortex	–	–	–	60, −18, 26	5.84	0.001
MMN	Parietal cortex (BA7)	−28, −68, 52	8.11	<0.001	12, −68, 52	9.07	<0.001
	Auditory cortex	−62, −8, 16	6.80	<0.001	56, −14, 16	7.26	<0.001
	Sensory cortex	−50, −20, 44	7.10	<0.001	50, −22, 52	7.10	<0.001
	Premotor cortex / SMA (BA6)	−32, −18, 60	6.89	<0.001	20, −16, 72	6.80	<0.001
	Motor cortex	−62, −8, 16	6.80	0.001	–	–	–
	Frontal cortex (BA8)	−46, 20, 34	6.62	<0.001	40, 16, 42	6.16	<0.001
	Sensory cortex	−22, −30, 58	6.55	<0.001	–	–	–
	Inferior temporal lobe (BA21)	−60, −14, −24	6.16	<0.001	–	–	–
	Occipital lobe (BA19)	−16, −82, 40	6.06	0.003	–	–	–
	Premotor cortex / SMA (BA6)	−20, −4, 66	5.90	<0.001	–	–	–
	Parahippocampal gyrus	–	–	–	24, −6, −32	5.73	0.002
	Parietal cortex (BA40)	–	–	–	54, −38, 30	5.34	0.007
	Parietal cortex (BA40)	−58, −44, 32	4.82	0.006	58, −40, 28	5.29	0.008
	Parietal cortex (BA7)	−26, −78, 44	5.30	0.008	6, −56, 64	5.31	0.007
	Parietal cortex (BA40)	–	–	–	52, −36, 32	5.28	0.008
*W. > B*.	Premotor cortex / SMA (BA6)	−32, −18, 60	2.83	0.003^†^	30, −14, 56	3.02	0.002^†^

*The t-maps were calculated with SPM8. The Standard condition shows locations activated in response to the standard notes, the MMN condition the locations estimated from the MMNm difference waveforms, and the W. > B. condition shows possible locations where the MMNm responses to deviants on odd metric positions are stronger in the Western group than in the Bicultural group. BA, Brodmann area; SMA, supplementary motor area. The coordinates refer to the MNI RAS system in millimeters. P-values are corrected for Family-Wise Error at cluster-level, except p-values marked by †, which are shown uncorrected at peak-level*.

## Discussion

In our study we wished to find if differences in musical cultural listening background, behaviorally represented by surprise ratings, might be represented by field changes using the inattentive and automatic MMNm. We found that listeners with exposure to mainly Western music reported higher surprise ratings and higher MMNm amplitude when listening to attenuated tones on odd-numbered metric positions, which are usually accented in Western music, compared to the group with a bicultural musical background. Our findings suggest that lifetime exposure to musical cultural backgrounds creates expectations toward rhythm and meter possibly based on an engrained mental pattern of structure learned implicitly through music exposure.

Previous experiments on music perception have showed that attenuation or omission of a metrically accented beat of binary or ternary meters consistently elicits an MMN with higher amplitude in Western listeners, in comparison to a deviant on a metrically weak beat (Vuust et al., 2005; Abecasis et al., 2009; Honing and Ladinig, 2009; Geiser et al., 2010; Bouwer et al., 2014). However, the previously reported effects of metric position on latency are inconsistent. The odd deviant has sometimes been observed as slower (Geiser et al., 2010; Bouwer et al., 2014) or faster (Martin et al., 2007; Ladinig et al., 2009) in latency compared to the even deviant. Our present findings replicate the previous observation of changes in the amplitude by showing that an MMNm with higher amplitude is elicited in listeners exposed to Western music when they hear an odd deviant. This supports that listeners habituated to Western music styles expect to hear strong accents on odd-numbered beats, which are frequently accented in the binary meters (cf. Fraisse, 1978; Brochard et al., 2003; Huron, 2006, p. 195; Potter et al., 2009). Also, the results suggest that a bicultural group of listeners with additional habituation to traditional Sub-Saharan African music styles, where regularly accented tones might be less common (cf. Arom, 1989), show lower expectations toward strong accents on odd-numbered tones. We also observed a shorter latency of the MMNm in the bicultural group compared to the Western group. This finding might be related to the fact that meters in certain Sub-Saharan African music are more complex compared to meters in popular Western music (Arom, 1989; Eerola et al., 2006; Hannon et al., 2012b). Since a short MMN latency is commonly associated with high perceptual abilities, the present finding might suggest that exposure to Sub-Saharan African music improve the perception of metric accents. Though, it must be taken into consideration that previous findings on MMN latency to metric deviants were less consistent compared to the latency of MMN responses to other types of deviants. It would be relevant in future studies to investigate whether a shorter MMN latency could be related to improvements in perception of metric accents in bicultural music listeners compared to monocultural Western music listeners, e.g., due to benefits in auditory perception related to specific effects of exposure to certain complex meters or general effects of culturally diverse music exposure in bicultural music listeners.

Because it has been shown that expert jazz musicians and trained percussionists elicit MMN responses with higher amplitudes and shorter latencies to metric deviants in comparison to non-musicians (Vuust et al., 2005, 2012; Geiser et al., 2010), we wanted to control for musical expertise using the MET as an important covariate. However, we did not find that amplitude and latency of the MMN was influenced by musical training. This finding is supported by another experiment (Bouwer et al., 2014). The lacking influence of rhythm perception ability in the present study on the MMNm amplitude and latency suggests that differences in rhythmic ability did not disturb the present findings of effects of enculturation on the MMNm. The missing influence of variations in rhythm perception ability might be explained by the relatively homogeneously high MET rhythm scores for the participants in the present experiment. Alternatively, the lacking effect of variation in the MET scores might be related to differences in the stimuli applied across experiments. Certain auditory deviants could be perceived as violations of expectations learnt through passive exposure to music, so-called “schematic expectations,” which both musicians and non-musicians are able to perceive equally well (cf. Ladinig et al., 2009), whereas other musical deviants could be perceived as performance errors, or violation of veridical expectations, which actively trained musicians would detect better than untrained non-musicians (Huron, 2006, p. 224f; Rohrmeier and Koelsch, 2012). This distinction between schematic violations and performance errors might explain why the stimuli in some experiments elicit different MMN responses in musicians in comparison to non-musicians. However, there is a need for a clear definition of which musical deviants might be interpreted as schematic violations or performance errors, and future investigations would be necessary to show whether they may influence the MMN differently in non-musicians and musicians.

The MMN has mainly been observed in the auditory cortex (Näätänen et al., 2001, 2007, 2010; Garrido et al., 2009), and the medial geniculate in the thalamus and inferior frontal gyrus have also been related (also see Atienza et al., 2002; Pulvermüller and Shtyrov, 2006; Moore and Linthicum, 2007; Garrido et al., 2009; Lai et al., 2011; Musacchia et al., 2014). In our study the estimated source locations of the metric MMNm are overall consistent with previous observations. The topographical distributions of the metric MMNm on the MEG sensors show common bilateral dipole fields around the temporal lobes in the axial magnetometers (cf. Scharinger et al., 2012) and typical field changes above the temporal lobes in the planar gradiometers. We observed source estimates for the metric MMNm, which are consistent with the previous MRI findings of increased gray matter density in the inferior temporal lobe, left motor cortex (Chakravarty and Vuust, 2009), and premotor cortex (Bailey et al., 2014) related to increased skills in rhythm and meter perception. Together, these findings suggest that the inferior temporal lobe, left motor cortex, and premotor cortex are involved in meter perception and in eliciting the observed metric MMNm. Also, they suggest that the metric MMNm originates in regions of the cortex, which are subject to plastic changes through active or passive music exposure. In addition, the estimated involvement of the right parahippocampal sources for the metric MMNm replicates previous MEG source modeling findings related to meter perception (Fujioka et al., 2010; James et al., 2012), suggesting an additional memory-related component involved in eliciting MMNm to metric deviants. Therefore, the hippocampus may be involved in the consolidation of the learned meter patterns due to exposure to a musical cultural background. The observed source estimate for the evoked responses to the standard tones located in the inferior frontal cortex (BA 47) in the present study is furthermore consistent with previous fMRI findings on motor-perceptual motor tasks involving tracking the meter in music (Levitin and Menon, 2003; Vuust et al., 2006; Ono et al., 2015).

Finally, longitudinal brain changes related to rhythm and meter processing could also be present in other domains and brain areas. Therefore, it would be relevant also to investigate whether brainstem responses (Tierney and Kraus, 2013), thalamic responses (Musacchia et al., 2014), N1 and P2 components (Abecasis et al., 2009; Schaefer et al., 2011; Vlek et al., 2011) and oscillatory responses (Snyder and Large, 2005; Zanto et al., 2006; Iversen et al., 2009; Fujioka et al., 2015) to metric accents are affected by exposure to music of different cultures.

## Limitations

The present sample size was relatively small, though the possibility of measuring cultural samples by applying high density MEG channel arrays is relatively rare due to the immobility of the MEG system. Also, participants with exposure to traditional Sub-Saharan music genres are rare to recruit within minor European cities outside the African continent. In this respect the present data set is relatively rare. The present numbers of 12–13 participants per group is comparable to the average 11 (range: 10–12) in previous cognitive neuroscience studies using the same stimulation paradigm (Brochard et al., 2003; Potter et al., 2009) and the average 10 (range: 5–14) in previous cognitive neuroscience studies comparing groups of listeners with exposure to non-Western and Western music (Neuhaus, 2003; Nan et al., 2006; Demorest et al., 2009). Another related issue is that the interpretation of the influence of exposure to Sub-Saharan African music and Western music on the MMNm responses to metric deviants is not straightforward. Anthropologists have argued that it may be appropriate to interpret the term “culture” as patterned social practices (Roepstorff et al., 2010). As such Sub-Saharan African music and Western music are heterogeneous concepts with regard to the diversity of “Sub-Saharan African” and “Western” music genres, and thus they refer to more than two homogenous “Sub-Saharan African” and “Western” music genres. It would be interesting to see whether in future studies applying mobile EEG it might be possible to achieve larger sample sizes and to perform measurements on meter perception in more specific cultural settings, such as in specific Ghanaian or a Central African locations. Though, MEG systems can achieve higher signal-to-noise ratios than EEG, which is a relevant aspect to consider if the measured effect size of enculturation is relatively small, in particular if the relatively subtle MMN response is measured. The influence of exposure to rhythms in languages of different cultures observed in behavioral studies (Iversen et al., 2008; Yoshida et al., 2010; Hay et al., 2011; Roncaglia-Denissen et al., 2013) could also be further investigated in the context of MMN paradigms. Although in the present MMN experiment the native language of the majority of participants was Danish, and on average the participants of both groups had been living in Denmark for about 2/3 of their life, it is likely that exposure to rhythmic structure in languages of different cultures can affect the MMN response to rhythmic stimuli. In our experiment we investigated the influence of exposure to bicultural musical background and Western music on surprise ratings and MMNm responses to attenuated tones at specific metric positions. In this case, it is relevant to apply ambiguous isochronous sinus tone sequences, otherwise ecologically realistic rhythms would have distorted the effects of exposure on the MMNm component, because the participant would be able to form expectations of metric accents based on variations in sound features of the ecological stimuli (cf. Bouwer et al., 2014). Though, it has been found that applying ecological stimuli improves listeners' detection of metric accents (Bolger et al., 2013) and might enhance the amplitudes of the brainstem wave V and cortical P1 components in response to metric accents (Tierney and Kraus, 2013; Bouwer et al., 2014). Therefore, although the simple ambiguous sinus tone sequences are important for the interpretation of the present experiment, these simplified stimuli may also weaken the MMNm amplitude in response to metric deviants in comparison to previous experiments applying more ecological stimuli (cf. Bouwer et al., 2014). Also, the relatively strong alpha waves in this experiment, mainly disturbing the magnetometers, could be related to a lower level of attention to the stimuli and increased introspective resting state processes when listening to the simplified stimuli compared to ecological stimuli with richer detail. In this respect, it is unclear whether the occipital-parietal source estimates in the present study were related to either alpha waves or to reorientation of attention in response to the unexpected metric accent deviants involving occipital brain regions. However, we applied a distributed source modeling approach, which enables analyzing occipital sources separate from more well-known brain regions related to rhythm and meter processing. It is possible that the ~10 Hz oscillations are mu waves associated with motor-related activity in the premotor cortex. Though, the gradiometer data did not show alpha or mu waves, as can be observed in the flat baseline preceding the MMNm in the gradiometers (Figure 4 top panel), which suggests that the alpha or mu waves did not influence the results.

## Conclusion

In our study, we investigated the effects of music enculturation on surprise ratings and MMNm brain responses to violations in metric deviants in Western and bicultural music listeners measured with MEG. Our results showed that listeners with a history of exposure to mainly Western music show stronger MMNm amplitude and higher surprise in response to attenuated odd numbered metric positions compared to the bicultural group. Also, we showed that the bicultural group appeared to discriminate less between metric positions in terms of surprise and showed shorter MMNm latency than the Western listener group. The neural generators of the observed metric deviant MMNm was estimated to be located in auditory, temporal, sensory-motor, and frontal brain regions, which are known to be involved in rhythm and meter perception and to increase in gray matter density through music training.

## Author contributions

All authors, NH, PV, FB, and EG-V, have contributed to the conception of the work, taken part in drafting and revising the manuscript, approved the final version of the manuscript, and agreed to be accountable for all aspects of the work.

### Conflict of interest statement

The authors declare that the research was conducted in the absence of any commercial or financial relationships that could be construed as a potential conflict of interest.
